# A Review of Current Knowledge on *Staphylococcus agnetis* in Poultry

**DOI:** 10.3390/ani10081421

**Published:** 2020-08-14

**Authors:** Gustaw M. Szafraniec, Piotr Szeleszczuk, Beata Dolka

**Affiliations:** Department of Pathology and Veterinary Diagnostics, Institute of Veterinary Medicine, Warsaw University of Life Sciences—SGGW, Nowoursynowska 159c St., 02–776 Warsaw, Poland; piotr_szeleszczuk@sggw.edu.pl

**Keywords:** *Staphylococcus agnetis*, poultry, broiler chicken, chondronecrosis, osteomyelitis, bovine mastitis

## Abstract

**Simple Summary:**

This literature review provides a synthesis and evaluation of the current knowledge on *Staphylococcus agnetis* (*S. agnetis*) and its implications in poultry pathology. Recent studies revealed that *S. agnetis* can cause bacterial chondronecrosis with osteomyelitis (BCO), endocarditis, and septicemia in broiler chickens. Lameness constitutes one of the major health and welfare problems causing huge economic losses in the poultry industry. To date, a range of infectious and non-infectious factors have been associated with lameness in poultry. Among bacteria of the genus *Staphylococcus*, *Staphylococcus aureus* is the main species associated with locomotor problems. This contrasts with *S. agnetis*, which until recently had not been considered as a poultry pathogen. Previously only reported in cattle, *S. agnetis* has expanded its host range to chickens, and due to its unique characteristics has become recognized as a new emerging pathogen. The genotypic and phenotypic similarities between *S. agnetis* and other two staphylococci (*S. hyicus* and *S. chromogenes*) make this pathogen capable of escaping recognition due to misidentification. Although a significant amount of research on *S. agnetis* has been conducted, many facts about this novel species are still unknown and further studies are required to understand its full significance in poultry pathology.

**Abstract:**

This review aims to summarize recent discoveries and advancements regarding the characteristics of *Staphylococcus agnetis* (*S. agnetis*) and its role in poultry pathology. *S. agnetis* is an emerging pathogen that was primarily associated with mastitis in dairy cattle. After a presumed host jump from cattle to poultry, it was identified as a pathological agent in broiler chickens (*Gallus gallus domesticus*), causing lameness induced by bacterial chondronecrosis with osteomyelitis (BCO), septicemia, and valvular endocarditis. Economic and welfare losses caused by lameness are global problems in the poultry industry, and *S. agnetis* has been shown to have a potential to induce high incidences of lameness in broiler chickens. *S. agnetis* exhibits a distinct repertoire of virulence factors found in many different staphylococci. It is closely related to *S. hyicus* and *S. chromogenes*, hence infections caused by *S. agnetis* may be misdiagnosed or even undiagnosed. As there are very few reports on *S. agnetis* in poultry, many facts about its pathogenesis, epidemiology, routes of transmission, and the potential impacts on the poultry industry remain unknown.

## 1. Introduction

The genus *Staphylococcus* belongs to the phylum *Firmicutes*, class *Bacilli*, order *Bacillales*, family *Staphylococcaceae*. As new research emerges, systematic relations within the genus *Staphylococcus* are constantly changing. Almost 60 *Staphylococcus* species have been identified, three of which were described as recently as last year (2019). New findings may cause some strains to be reclassified, as has been done for isolates previously identified as *Staphylococcus intermedius*. Most of these isolates are now recognized as *Staphylococcus pseudintermedius*. Together with *Staphylococcus delphini*, these three hard-to-differentiate species form a distinct *S. intermedius* group (SIG) [1].

Bacteria of the genus *Staphylococcus* are characterized by spherical, Gram-positive cells that occur singly, in pairs, or in small clusters. With a few exceptions, they are facultative anaerobes that are usually catalase-positive and oxidase-negative [2]. Staphylococci are ubiquitous in the poultry farm environment and belong to the normal bacterial microbiota of the skin and mucous membranes of healthy birds, including poultry and pigeons [1,2,3]. However, some species can cause opportunistic infections (Table 1). Such infections are common in poultry and are mainly caused by *Staphylococcus aureus*, the species most frequently isolated from birds diagnosed with staphylococcosis and the most pathogenic *Staphylococcus* species [3,4,5,6]. Locations from which *S. aureus* has been most commonly isolated include the proximal femur, proximal tibiotarsus, tendon sheaths, hock joints, pododermatitis lesions, heart, and liver [3,6,7,8,9,10,11,12,13,14]. Diseases promoted by staphylococci are often chronic in nature. Clinical symptoms and lesions vary with the site of entry of the pathogen and are primarily associated with bones, joints, and tendon sheaths; and less often with skin, heart, vertebrae, and other locations. Septicemia can develop following skin or mucous membrane infections. The most common findings in sick birds are lameness and fever, which can be followed by depression and death [3,6,7,15].

Staphylococcal infections are a global welfare and economic problem in poultry production. Economic losses are largely associated with lameness and its consequences, such as decrease in production parameters, increase in mortality, culling, and condemnation of carcasses at slaughterhouses. Staphylococcosis also contributes to reduced poultry welfare due to pain, stress, and decreased locomotive abilities [3,6,16,17,18].

Other noteworthy infectious agents associated with lameness in poultry include *Enterococcus* spp., *Streptococcus* spp., avian reovirus, Marek’s disease virus (MDV), chicken infectious anemia virus (CIAV), and infectious bursal disease virus (IBDV) [6,19,20,21,22,23]. Other than infections, there are also non-infectious factors that can cause abnormal skeletal development leading to lameness. Nutritional or metabolic imbalances, such as vitamin D deficiency, as well as phosphorus and calcium imbalances, have been associated with rickets, osteoporosis, and tibial dyschondroplasia [24,25,26,27,28]. Management practices and housing conditions, such as the bedding type, litter condition, lighting program, and stocking density, have been linked to higher incidences of leg disorders [29,30,31,32,33,34]. Previous research has highlighted associations between genetics, breed line, gender, growth rate, age, and leg problems in broiler chickens [16,31,35,36]. For many decades, genetic selection of broiler chickens was focused on improving the feed conversion rate and promoting rapid growth of chickens. Nowadays, broiler chickens can grow to 1.5 kg of body weight before they reach 30 days of age, while in the 1950s, reaching such weight would take 120 days [16,37]. Fast-growing chickens have higher nutritional requirements, which when not met, can lead to skeletal deformities. Additionally, their bones have less time to mature to be able to carry the excessive body weight [37]. The leg bone tissue of fast-growing chickens is more porous and less mineralized compared with that of slower-growing chickens, which makes fast-growing chickens more prone to bone deformities and leg trauma leading to lameness and pain [37,38,39]. In recent years, broiler selection programs have started to put more emphasis on improving health and welfare issues, such as skeletal integrity, cardiovascular fitness, and immunity [16].

## 2. Discovery and Identification of *Staphylococcus agnetis*

The first isolation of *S. agnetis* was performed from milk samples of subclinical and mild clinical intramammary infections in dairy cattle in southern Finland [49]. It was noticed that most isolates identified by the API^®^ Staph ID 32 test (bioMérieux, France) as *S. hyicus* did not cluster with any strain of known *Staphylococcus* species using amplified fragment length polymorphism (AFLP), which led to identification and description of this novel species. It was named in honor of Agnes Sjöberg (Finland, 1888–1964), who was the first female veterinary surgeon in Europe and the first woman in Europe with a doctoral degree in veterinary medicine (1918) [49]. Since then, *S. agnetis* has been isolated from cattle worldwide [50,51,52,53,54,55,56,57,58]. Later, it was discovered that *S. agnetis* is a causative agent of disease in broiler chickens and broiler breeders [40,41].

**Figure 1:**
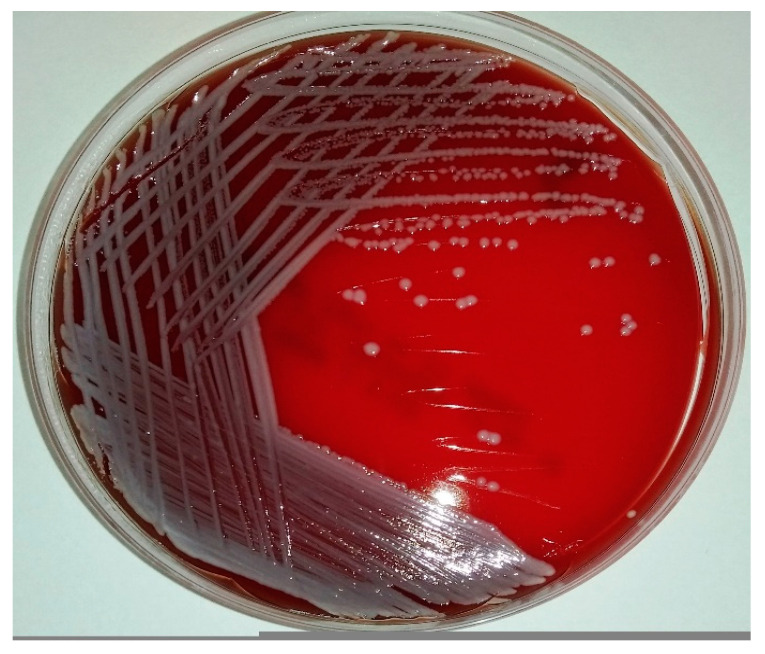
Colonies of *S. agnetis* (GenBank: MT231940) on Columbia agar with 5% sheep blood.

*S. agnetis* is a Gram-positive, coagulase-variable, facultatively anaerobic bacterium. It belongs to non-motile, non-spore-forming cocci, which occur singly, in pairs, or in small clusters. On 5% bovine blood agar after 18–24 h aerobic incubation at 37 °C, the bacterium forms smooth, circular to irregular, slightly convex, opaque, light grey, and non-hemolytic colonies growing up to 2–3 mm in diameter [41,49] (Figure 1).

Material for bacteriological tests should be collected aseptically from birds and plated on agar medium. Locations from which *S. agnetis* cultures have been successfully obtained include the proximal heads of the tibia and femur (either macroscopically normal or with BCO lesions), blood, liver, spleen, endocardium, and cloaca [40,41]. Basic media for staphylococcal samples are 5% blood agar (bovine or ovine blood), Müller–Hinton, or Trypticase Soy agar supplemented with blood [3,41,49,56]. In some cases, CHROMagar Orientation (DRG International, Union County, NJ, USA) has been used as a medium to initially differentiate cultured colonies [40,59].

Identification of *S. agnetis* and its differentiation from other staphylococci can be problematic. The morphologies of *S. agnetis* and *S. hyicus* colonies on blood agar are similar, which can lead to misidentification of these two species [41]. *S. aureus* colonies have yellowish pigmentation and are β-hemolytic or not hemolytic (γ-hemolytic) [3,41,49].

*S. agnetis* falls into the group of coagulase-variable staphylococci, with studies reporting isolates that could be either predominantly coagulase-negative [49] or coagulase-positive [54]. Some strains exhibited a delayed coagulase reaction in a tube test, with 12–25% of strains being found to be coagulase-negative after 4 h but coagulase-positive after 24 h. Clumping factor has been found to be negative [49,54], while the DNase test has been found to be positive [49].

Generally, biochemical tests (Table 2) can be used to differentiate between staphylococci [3,60]. However, they are a poor choice for *S. agnetis* identification. In addition, commercial biochemical identification systems (e.g., API^®^) cannot be used for identification of *S. agnetis*, as the species is not included in the comparison databases [61]. According to Taponen et al. [49], *S. agnetis* is very closely related to *S. hyicus* and *S. chromogenes* with regard to biochemical features. The authors demonstrated that within *S. agnetis* isolates, all but seven reactions gave identical results. The variable reactions were observed for glycogen utilization, β-glucuronidase, and acid production from D-galactose, amygdalin, melibiose, trehalose, and gentiobiose. However, not one of these reactions can be relied upon to distinguish *S. agnetis* from *S. hyicus* and *S. chromogenes*, as there are no instances of positive versus negative results between these species. Furthermore, tests for most of the differentiating reactions are not included in standard commercially available biochemical kits, making proper species identification even less likely. One study demonstrated that most staphylococci isolated from bovine milk, previously classified as coagulase-positive *S. hyicus* using phenotypic identification methods, were in fact not *S. hyicus* [54].

Matrix-assisted laser desorption/ionization–time of flight mass spectrometry (MALDI-TOF MS) has been used to confirm *S. agnetis* in poultry isolates after the type strain CCUG 59,809 of *S. agnetis* was added to the MALDI-TOF MS database as a reference [41]. However, MALDI-TOF MS and some genetic identification methods, such as 16S rDNA or *dnaJ* (heat shock protein 40, Hsp40) gene sequencing, were not sufficiently accurate in differentiating *S. agnetis* from other *Staphylococcus* species in some studies [54,61]. Gene sequencing of the β subunit of RNA polymerase (*rpoB*) and the elongation factor Tu (*tuf*) can be used to differentiate *S. agnetis* and *S. hyicus* when using sufficiently high cut-off values [54,56]. Real-Time PCR based on the cytochrome d ubiquinol oxidase subunit II (*cydB*) gene may provide a new approach to complement MALDI-TOF MS and other methods for the rapid and accurate identification of *S. agnetis* and coagulase-negative staphylococci. All of the isolates that were classified as *S. hyicus* by MALDI-TOF MS were identified as *S. agnetis* by using *cydB* real-time PCR [62].

Taponen et al. [49] constructed phylogenetic trees based on the 16S rDNA, *rpoB,* and *tuf* genes of 13 cattle *S. agnetis* isolates, as well as type strains of the genus *Staphylococcus*. The *S. agnetis* strains clustered closely together, indicating they belonged to a single species. They were distinct from *S. hyicus* ATCC 11,249 and *S. chromogenes* ATCC 43764, their closest relatives. An amplified fragment length polymorphism (AFLP) analysis using the restriction enzymes *Hind*III–*Mse*I and DNA–DNA hybridization (at a threshold value of 70%) proved to be useful tools for the differentiation of *S. agnetis* from *S. hyicus* and *S. chromogenes* [49]. *S. agnetis* can be identified to the species level by comparison of AFLP fingerprints with a large staphylococcal library or by a combination of *rpoB* gene sequencing and AFLP clustering [63].

A phylogenetic tree based on the 16S rDNA gene sequences of chicken *S. agnetis* (strain 908) and other staphylococci shows that *S. agnetis* clusters closely with *S. hyicus* and *S. chromogenes* [40]. *S. hyicus* is an etiological factor for swine exudative epidermitis and is frequently isolated from bovine mastitis [54,64]. In poultry, it is a common skin commensal bacterium known to cause opportunistic infections (Table 1). *S. chromogenes* is a common finding in cases of dairy cow mastitis [57,65]. In poultry, it is considered as a normal inhabitant of the skin and mucous membranes [64,66], although it has been reported to cause opportunistic infections [14] (Table 1). *S. hyicus* and *S. chromogenes* were not considered as separate species before 1986 [66], and similarly *S. agnetis* has only recently been acknowledged as a separate species from *S. hyicus* [49]. It should be noted that most cases of opportunistic infections caused by *S. hyicus* in poultry (Table 1) had been reported before *S. agnetis* was recognized as a distinct species. There is a possibility that *S. agnetis* diverged from *S. hyicus* a long time ago, and some of the infections, which back then were diagnosed as *S. hyicus* infections, might have been caused by *S. agnetis*. Adkins et al. [54] theorized that misidentification of species of coagulase-positive non-aureus *Staphylococcus* species may be a widespread phenomenon. In their study, genetic methods based on selected housekeeping genes were proven to be the most accurate and were used to decisively confirm whether the isolated species was *S. hyicus* or *S. agnetis*. Re-testing the cattle isolates, designated by phenotypic test (API^®^ Staph; bioMérieux, Lyon, France) as *S. hyicus,* using *tuf* gene sequencing led to their identification as *S. agnetis* (69.4%, 43/62), *S. chromogenes* (12.9%, 8/62), and *S. aureus* (8%, 5/62), with only 1.6% (1/62) confirmed as *S. hyicus*. The remaining 8% (5/62) were of various other *Staphylococcus* species. To better differentiate between *S. hyicus*, *S. agnetis*, and *S. aureus*, a method using the multiplex PCR assay combined with pulsed-field gel electrophoresis (PFGE) was proposed. In multiplex PCR, a partial segment of the *aroD* gene (3-dehydroquinate dehydratase) was amplified to identify *S. hyicus* and *S. agnetis*, while the *nuc* gene (thermonuclease) was amplified to identify *S. aureus* [54,56].

## 3. Role of *S. agnetis* in Poultry Pathology

*S. agnetis* has been primarily associated with bovine mastitis [49,51,52,53,54,55,57]. Since 2015 it has been identified as an etiological factor responsible for bacterial chondronecrosis with osteomyelitis (BCO) [40,59], endocarditis and septicemia in broiler chickens (*Gallus gallus domesticus*) [41] (Table 3).

### 3.1. Bacterial Chondronecrosis with Osteomyelitis (BCO)

Bacterial chondronecrosis with osteomyelitis (BCO) is a predominant cause of lameness in broiler chickens. Lameness is a significant cause of economic losses in the poultry industry, as well as a major concern for the welfare of the birds. In the 1970s, when BCO was first reported in Australia as a common cause of leg weakness in chickens, the incidence of lameness due to BCO in commercial broiler chicken flocks reached 50%, with up to 5% mortality of birds aged 4–8 weeks [68]. According to a later study, BCO was the most common cause of lameness and was found in 17.3% of lame chickens (33/191). Overall, BCO was diagnosed by histopathology in 13.7% (57/416) of the total dead and culled birds [6]. In another study, BCO in the proximal end of the femur and in the proximal end of the tibiotarsus was found in 20.4% of birds [7]. Overall, approximately 30% of commercially reared broiler chickens in the European Union have been reported to present leg abnormalities resulting in the decrease of locomotor functions [16,31,69]. According to Wijesurendra et al. [70], BCO occurs throughout the life of a broiler chicken flock at a very high rate, and the prevalence of histologically confirmed BCO is 28% (95% confidence interval (CI): 23–34%) of mortalities and culls. In addition to the welfare concerns, the annual economic losses due to leg problems for the poultry industry in the United States have been estimated at $80–120 million in broiler chickens and $40 million in turkeys [71]. In the UK, the overall losses associated with BCO were approximately 0.75% of male chickens of all bird placements, which cost the UK broiler industry £3 million annually. Another author found that BCO constitutes approximately 0.5–0.7% of the losses (through mortality and culling) from the total annual UK broiler production, which represents 3.75 million birds and costs £4.7 million [19]. Based on the above calculations, it has been estimated that 12.5 billion broiler chickens have leg problems worldwide per year [72].

BCO forms when the rapidly increasing weight of a growing chicken exerts excessive mechanical stress on epiphyseal and physeal cartilages, most notably of the proximal femur and tibia, creating osteochondrotic microfractures that can be colonized by hematogenously disseminated bacteria. These bacteria can form osseous sequestration, which may develop into necrotic lesions [23]. The first reported case of *S. agnetis* in poultry came from broilers experimentally reared on an elevated wire flooring [40], a model designed specifically to induce BCO and enhance its prevalence [23,73]. Although many different opportunistic bacteria (e.g., *S. aureus*, *Escherichia coli*, *Enterococcus cecorum*) were previously isolated from BCO lesions [23,40,73], in one study the majority (87.1%, 81/93) of isolates from BCO and blood of lame birds belonged to a single species—*S. agnetis* [40]. It was demonstrated that *S. agnetis* could be isolated from different sites of the same bird, and even from macroscopically normal bones. In those cases, bacteria were not cultured from blood samples, which indicated that bacteria in the bone did not originate from a generalized (systemic) infection [40]. One isolate out of 81 was designated as *S. agnetis* strain 908 and chosen by Al-Rubaye et al. [40] for detailed analyses and further studies [59].

It was confirmed that administration of live *S. agnetis* (strain 908) into drinking water induces high incidences of lameness (>50%) in broilers when combined with the wire flooring model [40,59]. Even though inoculation with *S. agnetis* happened early in the chicks’ lives, the first lame birds became identifiable only from day 35, which means that BCO lameness can be induced by exposing birds to *S. agnetis* at an early age [59]. This implies that *S. agnetis* 908 is able to persist in birds, possibly in the intestinal tract [23,59], until later in life when it enters the bloodstream and colonizes growth plates of the rapidly growing leg bones. The mechanism of survival of *S. agnetis* in the chicken intestines is unknown, however since *S. agnetis* shares some virulence determinants with *S. aureus* [40], it could be similar to mechanisms proposed and demonstrated in vitro for human *S. aureus* strains. Some authors showed that *S. aureus* can adhere to the mucus of the intestinal tract [74], while other demonstrated that *S. aureus* can be internalized by enterocytes and survive in this way for prolonged periods of time [75,76]. A model for BCO pathogenesis proposed by Wideman [23] is based on the ability of opportunistic pathogenic bacteria to translocate across the intestinal tract into the blood. Probiotics have the potential to combat enteral bacterial infections by means of competitive exclusion and other modes of action [77,78,79]. It has been demonstrated that the use of some probiotics in chickens raised on the wire flooring model can reduce the prevalence of lameness [73,80]. However, the same probiotics when administered in feed or water were unable to reduce the incidence of lameness when birds were challenged with the minimal effective dose of *S. agnetis* (strain 908). This shows that *S. agnetis* was able to overcome the protective properties of the probiotics used [59].

When birds were challenged simultaneously with *S. agnetis* (strain 908) and the human strain of *S. aureus* ATCC 27661, all cultures from BCO lesions yielded only *S. agnetis* [40]. This suggests either that *S. agnetis* is much more effective at colonizing growth plates and inducing BCO lameness than *S. aureus* ATCC 27661, or that this particular strain of *S. aureus* is unable to do so, which may be due to its lack of virulence determinants adapted to the chicken host [59,81,82]. Comparing chickens challenged with *S. agnetis* 908 and two other staphylococci isolated from BCO lesions (*S. saprophyticus* and *S. epidermidis*), authors showed that these two species induce lameness to a significantly lesser extent (cumulative % lameness: *S. saprophyticus* 57.4%, *S. epidermidis* 52.3%) than *S. agnetis* (80.5%) or no challenge (control group) (71.7%). The unchallenged control group yielded nearly as many lame birds as the group challenged with *S. agnetis* 908, which could indicate that the experimental environment of the research facility became heavily loaded with *S. agnetis* [59]. In addition, *S. agnetis* could have been transmitted from challenged to unchallenged birds in the same pen. The fact that challenges with *S. saprophyticus* and *S. epidermidis* resulted in lower degrees of lameness than in the group challenged with *S. agnetis* 908 (and the unchallenged group) could point towards their lower virulence for poultry. Furthermore, it may be assumed that *S. saprophyticus* and *S. epidermidis* have a potential protective effect against the acquisition of *S. agnetis* 908 from the environment. There may be ecological interactions between *S. agnetis* and other *Staphylococcu*s species that may be important factors for both colonization and BCO induction.

Al-Rubaye et al. [59] and Shwani et al. [83] pointed out that it is likely that *S. agnetis* 908 represents a hypervirulent clone that evolved from less virulent *S. agnetis* strains circulating in broiler populations at the research facility. The low level of genetic diversity has been observed between *S. agnetis* isolates retrieved from BCO chickens. Due to many years of passages and selective pressure on the wire flooring at the research facility, *S. agnetis* strain 908 became highly capable of inducing BCO lameness [59,83]. It seemed to be specific for that research facility.

### 3.2. Endocarditis and Septicemia

Valvular endocarditis is one of possible outcomes of infection with *Staphylococcus* spp. and other bacteria. It occurs when septicemic bacterial infection progresses to a subacute or chronic stage [84]. Endocarditis caused by *S. aureus* is well documented in humans [85,86], and has been reported in poultry [87]. *Staphylococcus simulans* has also been associated with endocarditis in broilers [88]. Bacteria of genera other than *Staphylococcus* that have been associated with natural or experimental poultry infections resulting in bacterial endocarditis include *Avibacterium endocarditidis*, *Enterococcus faecalis*, *E. faecium*, *E. hirae*, *E. durans*, *Streptococcus pluranimalium*, *S. gallolyticus*, *S. gallinaceus*, *S. zooepidemicus*, *Pasteurella multocida*, *Erysipelothrix rhusiopathiae*, and *Helcococcus ovis* [87,89,90,91,92,93].

The prevalence of bacterial endocarditis in poultry is not well described in the literature, with case studies reporting sporadic incidences that are usually limited to one flock or farm. According to reports from the 1960s [94], mortality due to endocarditis in a flock was less than 0.5%. Valvular endocarditis may be found in 15% of chickens older than 40 weeks of age, while in only 3% of chickens between 10 and 40 weeks of age. Out of 62% losses due to septicemia in broiler breeders, 34% of birds suffered from bacterial valvular endocarditis [87]. Increased mortality (17–20.1%) has also been observed in broiler breeder flocks, in which 29% of birds developed valvular endocarditis [95]. According to Velkers et al. [96], bacterial endocarditis may be responsible for 36% of the total mortality during the production of commercial broiler chickens. In other studies, endocarditis caused by *S. aureus* was noted in 5% of all affected birds [87], while that caused by *S. simulans* was noted in 40% of tested birds [88].

During longitudinal studies of broiler breeder mortality in four flocks in Denmark, Poulsen et al. [41] demonstrated *S. agnetis* to be an etiological agent for valvular endocarditis and septicemia in broiler breeders. *S. agnetis* was isolated in pure culture from 2.7% (*n* = 16) of all examined broiler breeders that died due to an infection. In these sixteen cases, isolates were obtained from the endocardium, liver, or spleen. Endocarditis was the primary cause of death in six hens (37.5%), four of which also showed signs of septicemia manifested as disseminated necrotic foci in the liver. In three cases (18.75%), septicemia with enlarged liver or spleen and circulatory shock was indicated as the cause of death. In the remaining seven cases (43.75%), hens died of other causes, but *S. agnetis* was isolated from the liver or spleen, and in one instance from the endocardium, despite the absence of endocarditis lesions (6.25%). It was assumed that in these seven cases, *S. agnetis* infections were secondary. It was speculated that *S. agnetis* could have a predisposition to injured endothelial cells, as has been shown in cases of human *S. aureus* endocarditis [41,97].

## 4. Genome Characteristics of *S. agnetis*

The first *S. agnetis* genome was characterized in 2014 [51]. The authors presented a draft genome sequence for an isolate obtained from a lactating dairy cow with subclinical mastitis. The genome of the mastitic isolate was approximately 2.42 Mbp. Al-Rubaye et al. [40] reported the first genome analysis of *S. agnetis* originating from poultry (strain 908). The estimated size of the genome of chicken *S. agnetis* was 2.47 Mbp. Recently, Shwani et al. [83] assembled a genome of *S. agnetis* 1416 isolate obtained from a commercial broiler farm and identified additional plasmids in poultry isolates [83].

When all predicted protein sequences were compared to other *Staphylococcus* spp. genomes (*S. hyicus*, *S. pseudintermedius*, *S saprophyticus*, and five *S. aureus* from public databases), it was found that the majority of *S. agnetis* 908 predicted proteins had their closest homologs in *S. hyicus* (87.1%) [40]. Homologs in other species were found for 5.1% of the predicted proteins, while 8.5% had no match in the database. The closest homologs other than those from *S. hyicus* were most numerous within *Bacillus* spp. and other *Staphylococcus* spp. When *S. hyicus* genome was excluded from comparison, the next closest homologs to *S. agnetis* 908 genes were found most frequently in canine *S. pseudintermedius*, followed by the human *Staphylococcus* member HGB0015 GN and human *S. aureus*. The assembled genome of *S. agnetis* 908 differs from the *S. hyicus* genome in at least five notable regions. These regions contain orthologs to genes from various Gram-positive and Gram-negative bacteria [40].

A comparison of the genomes of different poultry *S. agnetis* strains with that of *S. agnetis* 908 showed little to no diversity in the main chromosome. However, most of the differences occurred in the plasmid, making it hypervariable in *S. agnetis* isolates. [40]. After performing a BLASTN search on all three assembled plasmids of *S. agnetis* 908 against 26 *S. agnetis* genomes present in the NCBI database, Shwani et al. [83] concluded that none of these plasmids seems to harbor genes determining chicken host specialization.

Pulsed-field gel electrophoresis (PFGE) typing of the Danish isolates of *S. agnetis* from broiler breeders and newly hatched chickens showed that the isolates were divided into seven types based on the PFGE band patterns. In total, 29 isolates were tested. These were obtained from the liver (*n* = 10), spleen (*n* = 4), and heart (*n* = 2) of broiler breeders, as well as cloacal swabs of newly hatched chickens (*n* = 13). One PFGE type seemed to be predominant and included both isolates from septicemia in broiler breeders from three different farms, as well as isolates from newly hatched chicks derived from one of these farms. This could indicate that *S. agnetis* may be transmitted from breeders to their offspring [41].

## 5. Virulence Factors of *S. agnetis*

*S. agnetis* has a distinct repertoire of virulence factors with homologs to both pathogenic and non-pathogenic staphylococci. Virulence factors belonging to the classes of host immune evasion, host adherence, toxin biosynthesis, and secretion systems were identified. Among these, the most noteworthy were master regulatory virulence genes found in *S. aureus* [40].

Exotoxins produced by *S. agnetis* 908 consist of five superantigen-like proteins (also identified in the Danish isolates [41]), a β-hemolysin, and an exfoliative toxin A. When compared with some other *Staphylococcus* species (*S. hyicus*, *S. aureus*, *S. pseudintermedius*, *S. chromogenes*), they showed varied relatedness, sometimes contrary to what could be expected based on the 16S rDNA phylogenetic tree [40]. Exotoxins have been found to be crucial in the pathogenesis of staphylococcal infections, interacting with host cells and tissues in various ways [98,99,100,101].

Staphylococcal superantigen-like proteins (SSLs) are a group of virulence factors known for their ability to manipulate the host’s immune system [98]. One of the SSLs, SSL7, is able to bind immunoglobulin A (IgA), an important element of local mucosal immunity, contributing to the survival of staphylococci on the surface of mucous membranes, i.e., inside the gut [102,103]. Another function of SSL7 is inhibition of the activity of the complement, an important defense against staphylococcal infections, by binding to the complement factor C5 [102]. Other SSLs exhibit functions that include inhibition of neutrophils and macrophages, as well as binding of glycan, Fc receptors, and P-selectin [104,105,106,107,108].

The exfoliative toxin A gene (eta) harbored by the chicken strain *S. agnetis* 908 clustered most closely with the eta of *S. hyicus* [40]. It was also found in *S. agnetis* isolates from 75% (3/4) of mastitic milk samples [67], which was contrary to other studies in which only the etd gene (encoding exfoliative toxin D) was found among the exfoliatin genes tested (eta, etb, etd) [58]. Exfoliative toxins (ETs) are virulence factors mostly associated with *S. aureus* and S. hyicus infections [109,110,111,112]. They contribute to exfoliative epidermitis in swine and staphylococcal scalded-skin syndrome in humans. It has been shown that subcutaneous inoculation of some ETs can cause epidermis exfoliation in one-day-old chicks. However, inoculation with ETs A, B, and D originating from human *S. aureus* strains has shown no exfoliative activity in chicks [113,114,115]. It seems that ETs found in *S. agnetis* isolates have no effect on chickens, and to our knowledge there are no publications highlighting the role of ETs in poultry infections. The eta gene has not been found in poultry methicillin-resistant *S aureus* (MRSA) isolates, and only rarely (1.7%) in methicillin-sensitive *S. aureus* [116]. However, it should be noted that both *S. hyicus* and *S. chromogenes*, close relatives to *S. agnetis*, have been reported to produce ETs that can affect chicken epidermis [109,114,115,117].

Poulsen et al. [41] theorized that the fibronectin-binding proteins (FnBPs) of *S. agnetis* are crucial in its ability to cause endocarditis in broiler breeders, and that FnBPs allow *S. agnetis* to adhere to injured endothelial cells. The role of FnBPs in the pathogenesis of *S. aureus* endocarditis has been proven in mammals, including humans [97,118,119,120], but it remains to be confirmed in birds. The genome of chicken *S. agnetis* includes seven genes associated with FnBPs that play a role in cell adhesion [40,41]. The *S. agnetis* FnBPs appear to be distinct from those of *S. hyicus*, and seem to have been acquired after *S. agnetis* and *S. hyicus* diverged [40]. In the Danish strains of *S. agnetis*, one of the seven FnBPs can also be found in *S. hyicus*, while a mobile genetic element located upstream of the FnBP gene indicates a possible transfer of that gene from another staphylococcus [41]. Genes associated with FnBP production have also been found in *S. agnetis* isolates from mastitic milk in Finland and Canada [67,121].

## 6. Antimicrobial Resistance in *S. agnetis*

The antimicrobial resistance of *S. agnetis* has been studied in cattle isolates worldwide [49,50,53,58,122]. To our knowledge, there are no reports or data on antimicrobial resistance in poultry isolates of *S. agnetis*.

The mastitic *S. agnetis* usually show resistance to penicillin, ampicillin, cefotaxime, clindamycin, polymyxins, lysozyme (muramidase), and deferoxamine [49,53], although some isolates may be sensitive to benzylpenicillin and dicloxacillin [53,122]. It is worth mentioning that *S. agnetis* has exhibited methicillin resistance associated with the *mecA* gene (encoding an alternative penicillin binding protein—PBP-2α) [50,58]. However, some *mecA-*positive strains may be susceptible to oxacillin [58]. Resistance to oxacillin (also conferring resistance to methicillin) and fusidic acid has been found in most isolates [122]. Overall, *S. agnetis* isolates have exhibited susceptibility to erythromycin, tetracycline, aminoglycoside antibiotics, phenicols, ciprofloxacin, trimethoprim or sulfamethoxazole, vancomycin, cephalothin, gentamicin, novobiocin, lysostaphin, and bacteriocins produced by *Bacillus thuringiensis* [49,53,122].

Recently, the potential application of bacteriocin synthesized by *S. agnetis* has been discussed [123,124]. *S. agnetis* strain 3682 (previously *S. hyicus* 3682) was discovered to produce a bacteriocin, named agneticin 3682, that manifested a broad spectrum of antimicrobial action [123]. It was the first bacteriocin described in this staphylococcal species. The agneticin-producing isolate showed a marked inhibitory activity against multidrug- and methicillin-resistant *S. aureus* (MRSA). Agneticin 3682 may offer a new strategy to fight against clinical MRSA isolates [123]. Similarly, the *S. agnetis* strain 4S97B isolated from goat and sheep milk was reported to produce a bacteriocin able to inhibit the growth of *Listeria monocytogenes*. However, it was ineffective against the remaining bacterial strains tested [124]. Bacteriocins produced by poultry *S. agnetis* isolates have not yet been described in the available literature.

It should be noted that the available data do not reflect antimicrobial resistance in this staphylococcal species as a whole. *S. agnetis* isolates showed varied resistance to antimicrobial agents, which most likely mirror local production systems, flock management practices, and the use of antimicrobials [125].

## 7. Host Jump of *S. agnetis*

To determine phylogenetic relationships between the poultry and cattle strains of *S. agnetis*, phylogenetic trees were constructed [83]. Shwani et al. [83] compared the chicken *S. agnetis* isolates to those from cattle and identified coding sequences distinguishing isolate 908 from the cattle isolates. In total, 5 chicken (*S. agnetis* 908, *S. agnetis* 1416, and the three Danish isolates) and 31 cattle *S. agnetis* genomes were included. The results showed that the chicken and cattle strains were closely related with no obvious separation, but all chicken isolates clustered together. Based on these findings, Shwani et al. [83] suggested that all chicken isolates radiated from a recent single host jump, most probably from cattle to poultry. The dendrogram from BLAST NCBI showed the closest relationship of the U.S. chicken isolate (908) to a bovine isolate (12B) obtained from milk of a buffalo with mastitis in Argentina.

Genomic comparisons of chicken and cattle isolates were performed to identify any genes that could have promoted *S. agnetis* to switch from localized infections of the mammary gland in cattle to systemic infections in poultry. Of the identified genome regions or genes that showed a high similarity within the chicken isolates, but which were more distinct in the cattle isolates, none are recognizable as virulence determinants or as being capable of mediating tissue tropism. It was also determined that none of the plasmids of *S. agnetis* 908 showed any genes corresponding to chicken host specialization. However, it is possible that some plasmid sequences were picked after jumping to poultry [83].

The other known case of a *Staphylococcus* species expanding its host range to poultry was the human-to-poultry host jump of *S. aureus* [81]. In this example, host adaptation resulted in *S. aureus* acquiring mobile genetic elements from an avian-specific accessory gene pool (which included two prophages, two plasmids, and a staphylococcal pathogenicity island), losing some of its function of human disease pathogenesis by inactivation of some of the virulence factors and acquiring enhanced resistance to chicken heterophils. Murray et al. [82] noted several recombination events in 33 genes along the branch expanding to the poultry-specific cluster of *S. aureus*. A group of 47 genes was found most often within poultry isolates when compared with those of human isolates. Poultry isolates were more adapted to chicken hosts, showing enhanced growth at 42 °C, and showed more pronounced chicken erythrocyte lysis. However, no such changes justifying chicken host specialization were found in *S. agnetis* genome. Thus, it was concluded that the cattle-to-poultry host jump of *S. agnetis* was probably facilitated by small alterations in a few virulence-associated factors [83].

## 8. Transmission of *S. agnetis*

Since 16S rDNA sequences highly similar to *S. agnetis* have been found in sheep scab mites [126], it is possible that the mites or other ectoparasites act as vectors for the disease between cattle and poultry. However, attempts at finding any mites using standard methods in flocks infected with *S. agnetis* have failed [59].

Al-Rubaye et al. [59] demonstrated that *S. agnetis* could be transmitted between hens in the same pen. As it was possible to induce infection by administering *S. agnetis* in the drinking water—the probable routes of dissemination could be by direct contact, through nipple waterers, or by shedding from birds that had an early bacteremia. It was suggested that *S. agnetis* could be spread via aerosols [59]. Such a route of transmission has been proven to be possible in *S. aureus* infections [127].

It is possible that parent flocks can transfer *S. agnetis* to their offspring. In the study by Poulsen et al., 0.34% of cloacal swabs collected from newly hatched chicks originating from flocks with confirmed *S. agnetis* infections were positive for *S. agnetis*. Isolates from the chicks were of the same PFGE types as the ones found in the broiler breeder flocks they originated from. Eight flocks of broilers supplied from these broiler breeder flocks were followed for their first week of life. Birds that died during that time were examined, and in none of these birds was *S. agnetis* determined to be the cause of death [41].

Because of the lack of clinical trials, natural routes of *S. agnetis* transmission, reservoirs, and potential vectors remain unknown.

Generally, the occurrence of a staphylococcosis disease outbreak requires a pathogen in sufficient quantities, a mode of transmission, and a susceptible host. *Staphylococcus* breaks down host natural defenses by damage to the skin or mucous membranes, which creates an entry point for the pathogen. Immunosuppression is another way to impair host defenses. Stress; poor biosecurity or management conditions; diseases such as IBD, CIA, and MD; and other factors limiting the function of the host immune system can facilitate the outbreak of the disease [3]. Further studies are needed to confirm whether *S. agnetis* follows these mechanisms.

## 9. Conclusions

Locomotor problems are a major health concern for the poultry industry worldwide. Lameness results in poor performance and substantial economic losses. There are numerous etiological factors known to cause lameness in poultry. Some factors are non-infectious, including those associated with management, housing conditions (e.g., condition of the litter), and nutrition (e.g., calcium to phosphorus ratio, vitamin D deficiency). In addition to the above, there is a wide range of infectious agents responsible for inducing lameness, including both viral and bacterial diseases (most notably infections by *E. cecorum*, *S. aureus*, avian reovirus, or Marek’s disease virus). Concluding all of the research covered in this review, *S. agnetis* should be counted among all of the aforementioned agents of disease and recognized as a new emerging pathogen in poultry, not only as the causative agent of mastitis in cattle. *S. agnetis* has been linked to lameness in broiler chickens, as well as mortality due to septicemia and endocarditis in broiler breeders.

## Figures and Tables

**Table 1:** List of *Staphylococcus* species associated with infections in poultry.

Staphylococcus Species	Poultry Species and Production Type	Diseases	Reference
*S. agnetis*	Broiler chicken, broiler breeder	Bacterial chondronecrosis with osteomyelitis, endocarditis, septicemia	[40,41]
*S. aureus*	All poultry species and production types	Arthritis, synovitis, chondronecrosis, osteomyelitis, gangrenous dermatitis, bumblefoot, green liver–osteomyelitis syndrome, omphalitis, septicemia	[3,6,7,8,9,10,11,12,13]
*S. auricularis*	Broiler chicken	Systemic infection	[42]
*S. capitis*	Broiler chicken, laying hen, broiler breeder	Systemic infection	[14,42]
*S. carnosus*	Broiler turkey	Systemic infection	[14,42]
*S. chromogenes*	Broiler chicken, broiler turkey, laying hen, broiler breeder, waterfowl	Systemic infection	[14]
*S. cohnii subsp. urealyticus*	Broiler chicken, broiler turkey, laying hen, broiler breeder, waterfowl	Hock joint arthritis, systemic infections, scabby hip syndrome	[14,42,43,44]
*S. epidermidis*	Broiler chicken, laying hen, waterfowl	Chondronecrosis with osteomyelitis (BCO), scabby hip syndrome, systemic infection	[14,42,44]
*S. gallinarum*	Broiler chicken	Systemic infection	[42]
*S. hominis*	Broiler chicken	Chondronecrosis with osteomyelitis (BCO), systemic infections	[14,40,42]
*S. hyicus*	Broiler chicken, laying hen, broiler breeder, turkey	Systemic infections, fibrinopurulent blepharitis and conjunctivitis, mixed infections with fowl pox, stifle joint osteomyelitis, acantholytic folliculitis and epidermitis, pododermatitis	[11,14,45,46,47,48]
*S. intermedius*	Broiler chicken	Systemic infections, scabby hip syndrome	[42,44]
*S. lentus*	Broiler chicken, broiler turkey, broiler breeder, laying hen, waterfowl	Systemic infections, scabby hip syndrome	[14,42,44]
*S. lugdunensis*	Broiler turkey	Systemic infections	[14]
*S. saprophyticus*	Broiler chicken, broiler turkey, laying hen	Chondronecrosis with osteomyelitis (BCO), systemic infections	[14,40]
*S. schleiferi*	Broiler chicken	Systemic infections	[14]
*S. sciuri*	Broiler chicken, broiler turkey, waterfowl	Scabby hip syndrome, systemic infection	[14,44]
*S. simulans*	Broiler chicken, broiler turkey, waterfowl	endocarditis, systemic infection, scabby hip syndrome,	[14,42,44]
*S. warneri*	Broiler chicken, broiler turkey, laying hen	Scabby hip syndrome, systemic infection	[14,44]
*S. xylosus*	Broiler chicken, broiler turkey, broiler breeder, waterfowl	Chondronecrosis with osteomyelitis (BCO), systemic infections	[14,40,42]

**Table 2:** Comparison of selected tests or biochemical characteristics of *S. agnetis* with other *Staphylococcus* species.

Test or Characteristic		*S. agnetis*	*S. aureus*	*S. hyicus*	*S. chromogenes*
Coagulase production		+/−	+	+/−	−
Hemolysis		−	+	−	−
Catalase production		+	+	+	+
Oxidase production		−	−	−	−
DNase production		+	+	+	w
Clumping factor		−	+	−	−
Acid (aerobically) from:	GLU (D−glucose) ^a^	+	+	+	+
	FRU (D−fructose) ^a^	+	+	+	+
	MNE (D−mannose) ^a^	+	+	+	+
	MAL (D−maltose) ^a^	−	+	−	+/−
	LAC (D−lactose) ^a^	+	+	+	+
	TRE (D−trehalose) ^a^	+/− (58.3%)	+	+	+
	MAN (D−mannitol) ^a^	−	+	−	+/−
	XLT (xylitol) ^a^	−	−	−	−
	MEL (D−melibiose) ^a^	+/− (7.7%)	−	−	−
	RAF (raffinose) ^a^	−	−	−	−
	XYL (xylose) ^a^	−	−	−	−
	SAC (saccharose) ^a^	+	+	+	+
	MDG (methyl−αD−glucopyranoside) ^a^	−	+/− (2%)	+/− (2%)	−
	NAG (N−acetyl−glucosamine) ^a^	+	+	+/− (93%)	+/−
	D−galactose ^a^	+/− (69.2%)	+	+	+
	Amygdalin	+/− (7.7%)	ND	−	−
	Gentiobiose	+/− (7.7%)	−	−	−
NIT (nitrates reduction) ^a^		+	+	+	+
PAL (alkaline phosphatase) ^a^		−	+	+	+
VP (acetoin production) ^a^		−	+	−	−
ADH (arginine dihydrolase) ^a^		+	w+	+	+
URE (urease) ^a^		−	w+	+/−	+/−
LSTR (resistance to lysostaphin) ^a^		−	−	−	−
β−glucuronidase		+/− (53.8%)	+	+/−	−/+
Glycogen utilization		+/− (15.4%)	ND	ND	ND
References		[49,67]	[2,60]	[2,49,60,64]	

Note: +, positive; −, negative; +/−, variable; (*n*%), percentage of positive reactions; w, weak reaction; w+, positive to weak reaction; ND, test not determined; ^a^, test included in commercial API^®^ Staph (bioMérieux, France).

**Table 3:** A summary of current studies of *Staphylococcus agnetis* in poultry.

Year	Country	Brief Conclusions	Reference
2015	USA	*S. agnetis* can be isolated from the blood, femur, and tibia of lame chickens reared on elevated wire flooring. *S. agnetis* is determined to be an etiological agent of lameness associated with bacterial chondronecrosis with osteomyelitis (BCO). *S. agnetis* administered in the drinking water induces lameness. Whole genome analysis shows *S. agnetis* to possess virulence factors from many different staphylococci. Closest relatives to *S. agnetis* are *S. hyicus* and *S. chromogenes*.	[40]
2017	USA	Minimal effective dose and optimal time for *S. agnetis* administration in the experimental challenge model are determined. *S. agnetis* administered in the water to chickens at an early age can induce BCO lameness later in life and overwhelms the protective properties of some probiotics. *S. agnetis* is transmittable between chickens.	[59]
2017	Denmark	*S. agnetis* infection can cause broiler breeder mortality associated with endocarditis and septicemia in broiler breeders. *S. agnetis* is also found in cloacal microbiota of a small number of newly hatched chicks originating from afflicted farms. It is possible that *S. agnetis* can be transmitted from broiler breeders to their offspring.	[41]
2020	USA	Whole genome comparisons between chicken and cattle *S. agnetis* isolates show that *S. agnetis* most likely performed a single host jump from cattle to poultry. No identified genes are currently associated with chicken host specialization, meaning that it could have been facilitated by minute mutations in a few genes associated with virulence factors.	[83]
